# Sickle Cell Trait and Adverse Pregnancy Outcomes: Is There a Link?

**DOI:** 10.7759/cureus.28610

**Published:** 2022-08-30

**Authors:** Huda Buhusayyen, Hasan M Isa, Nahid Kamal

**Affiliations:** 1 Obstetrics and Gynecology, Salmaniya Medical Complex, Manama, BHR; 2 Pediatrics, Arabian Gulf University, Manama, BHR; 3 Pediatrics, Salmaniya Medical Complex, Manama, BHR

**Keywords:** bahrain, outcome, neonatal, maternal, sickle cell trait, pregnancy

## Abstract

Objective

The objective is to assess the overall prevalence of maternal and neonatal pregnancy-related complications, and to compare their frequency among women with sickle cell trait (SCT) and those with normal hemoglobin patterns to examine the association between SCT and maternal and neonatal outcomes.

Methods

A retrospective cross-sectional case-control study was conducted by reviewing medical data from the labor room registry book and electronic database. All pregnant ladies delivered in Salmaneya medical complex from January 1 to December 31, 2019 were included. Patients were divided into two groups based on the presence or absence of SCT. Adverse maternal and neonatal outcomes were compared between the two groups.

Results

Out of the 5,067 pregnant women reviewed, 934 (18.4%) were included (460 (49.3%) with SCT and 474 (50.7%) with healthy controls). Overall, maternal, and neonatal complications were noted in 40.8% (n=381) and 16.3% (n=152), respectively. In univariate analysis, patients with SCT were significantly more likely to underwent cesarean sections compared to healthy women, 28.7% (n=132) vs. 21.7% (n=103), respectively (P=0.044) and to have more intrauterine fetal death (3% [n=14] vs. 0.2% [n=1], respectively (P<0.0001). No significant differences were found between the two groups in terms of the occurrence of the pregnancy-related hypertensive disorder, gestational diabetes, small for gestational age, and preterm delivery.

Conclusion

The result of this large, retrospective cross-sectional, case-control study shows that pregnant women with SCT were associated with an increase in intrauterine fetal death in comparison with pregnant women with normal hemoglobin. There were no differences found in pregnancy-related hypertensive disorder, gestational diabetes, small for gestational age, and preterm delivery. This result will emphasize the requirement of additional studies to scrutinize these findings and to determine whether there may be a benefit of a unique antenatal surveillance guideline for such patients.

## Introduction

Sickle cell disease (SCD) is one of the most common hemoglobinopathies in Bahrain [1]. It is inherited as an autosomal recessive disorder characterized by chronic hemolysis and vaso-occlusive complications [1,2]. The incidence of parturient mothers affected with SCD in Bahrain between 2007 and 2012 was 0.55% [2]. SCD is associated with a significant adverse outcome for the mother and fetus [3]. SCD in pregnancy is linked to increased maternal mortality by six-folds, along with an increase in the risk of pregnancy-related hypertension disorders (PRHD), small for gestational age (SGA), preterm delivery (PTD), and intrauterine fetal death (IUFD) [4]. 

Controversially, sickle cell trait (SCT) influences on adverse maternal and neonatal outcomes are uncertain [5]. Several studies reported the association between SCT and adverse obstetric outcomes [6-9]. However, data are conflicting; some studies found a positive link between SCT and PRHD, SGA, and PTD [7-9]. In contrast, other studies showed no significant correlation between SCT and pregnancy outcome [6,10,11]. Therefore, the primary concern of genetic screening is focusing solely on avoiding offspring affected with SCD rather than the presence of SCT in the mother [6,11]. However, in patients with SCT, there is a possibility of participation in physiological changes that approach those seen in SCD especially if they are kept under cardiovascular strain, leading to an increase in morbidity and mortality [7,12]. Thus, based on this theory, SCT may affect the dynamic physiological processes of gestation and, therefore, negatively impacts pregnancy. If this theory is genuine, it could significantly influence preconception counseling and the antenatal follow-up inpatient with SCT. For instance, serial growth scan for screen and early detection of SGA, close blood pressure monitoring, prophylaxis low dose aspirin can be recommended to decrease the risk of PRHD, or intrapartum antibiotics prophylaxis might be given to reduce the risk of postpartum endometritis. Moreover, establishing such pregnancy risks could remarkably impact the current standard of care. Therefore, this study was conducted to assess the overall prevalence of maternal and neonatal pregnancy-related complications and to compare their frequency among women with SCT and those with normal hemoglobin patterns to examine the association between SCT and maternal and neonatal outcomes.

## Materials and methods

Study design and setting

A retrospective, cross-sectional, case-control study of women who delivered between January 1 and December 31, 2019 was conducted in the Department of Obstetrics and Gynecology and Department of Pediatrics, Salmaneya medical complex (SMC), Bahrain. 

Study population

**Figure 1:**
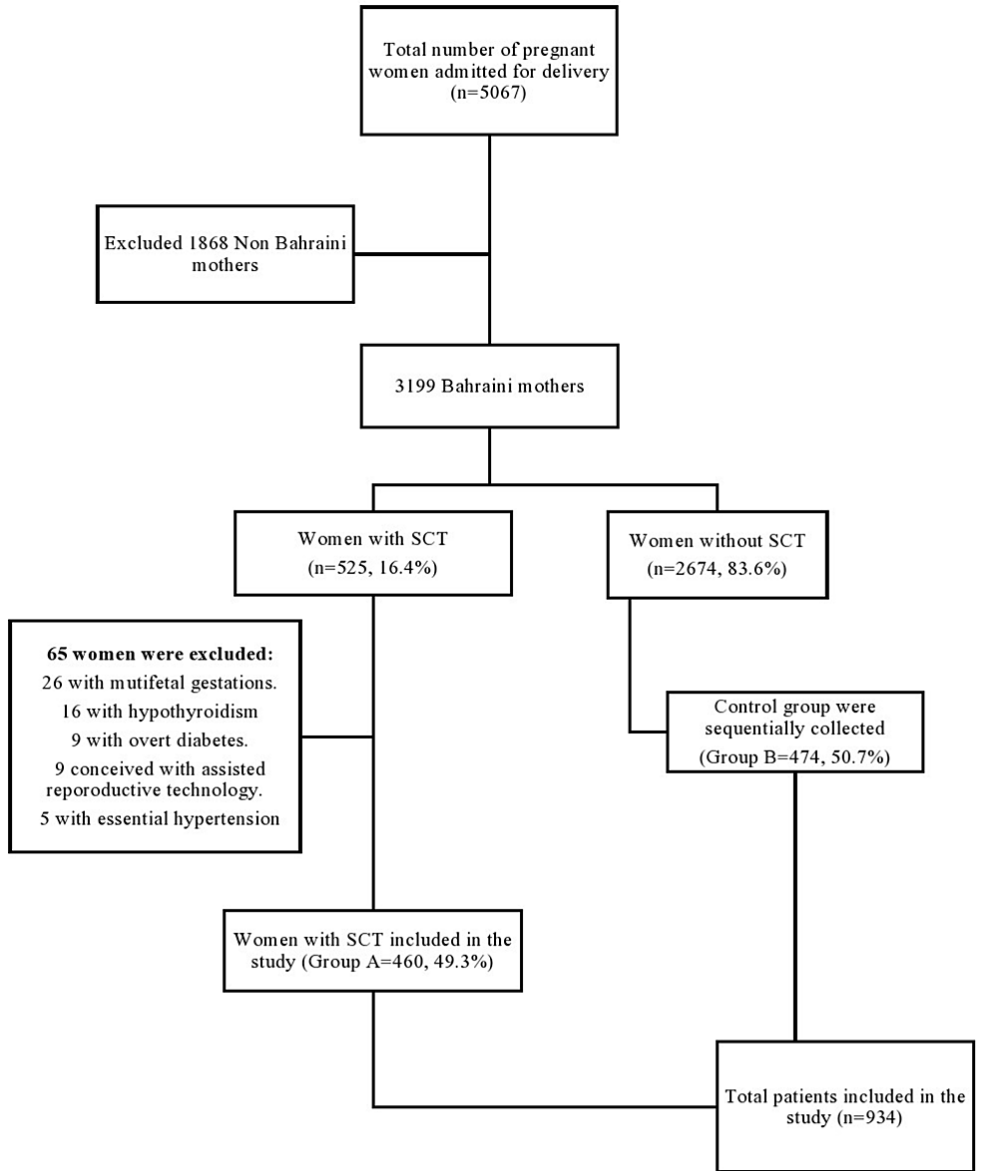
Flow chart summarizing the distribution of the data collection between January 1 and December 31, 2019. SCT: sickle cell trait

During the study period, all pregnant women admitted for delivery were divided into two groups based on the presence or absence of SCT, Groups A and B, respectively. Universal screening for hemoglobinopathy is performed as a standard of care for all pregnant women delivered in our department. Healthy pregnant women, found to have SCT were separated. Those who delivered before 24 weeks of gestation, had incomplete medical records, had multiple pregnancies, were non-nationals, and associated with comorbidities (overt diabetes, hypothyroidism, and essential hypertension) were excluded from Group A. A systematic selection of an aged-matched control group (Group B) including pregnant women with normal hemoglobin electrophoresis was performed sequentially until the end of the selection of group B patients list excluding any patient with similar exclusion criteria used in group A (Figure 1).

Data collection

Maternal and newborn data were collected from the labor room registry book and the electronic database (I-SEHA system). Mothers’ identification number, age, parity, gestational age at delivery, and mode of delivery were collected. Data about the adverse pregnancy outcomes for both groups were collected. The maternal outcomes included the development of PRHD and GDM. PRHD is defined according to the American College of Obstetrics and Gynecologists and subdivided into gestational hypertension, preeclampsia, eclampsia, and superimposed preeclampsia collectively [13]. GDM was diagnosed according to the American diabetes association criteria where one or more abnormal blood sugar readings (fasting blood sugar level ≥ 5.1 mmol/L, 1 hour ≥ 10 mmol/L, 2 hours ≥ 8.6 mmol/L) was detected with the use of 75 g, two hours glucose tolerance test [14].

The newborn's data include the sex of the infants, APGAR score, and weight at the delivery. Neonatal outcomes were IUFD (described according to the world health organization) [15], PTD (defined as delivery < 37 weeks of gestation), and SGA (defined as birth weight less than the 10th percentile) [16].

Statistical analysis

Data were analyzed using a statistical package for social science (SPSS) version 21 (IBM Corp., Armonk, NY, USA). Continuous variables were presented as means and standard deviation while categorical variables were presented as counts and percentages. The maternal two groups (with or without SCT) have been compared in terms of maternal and fetal outcomes. Mann-Whitney U test was used to compare continuous variables while Fisher’s exact test or Pearson’s Chi-square test were used to compare categorical variables. P-value < 0.05 was considered statistically significant.

## Results

Out of 5,067 women delivered in SMC during the study period, 3,199 (63.1%) were Bahraini, and 1,868 (36.9%) were not national. Five hundred twenty-five (16.4%) women with SCT were identified, all of them Bahraini. Out of the 525 patients, 65 (12.4%) were excluded - 26 (4.9%) with multifetal gestations, 16 (3%) with hypothyroidism, nine (1.7%) with overt diabetes, and those conceived with assisted reproductive technology each, and five (0.95%) with essential hypertension (see patients’ selection flow chart in Figure 1). The remaining 460 (49.3%) with SCT were included in Group A. Another 474 (50.7%) were allocated to Group B (normal hemoglobin) as healthy controls. The total number of both groups that were included in the study was 934 (18.4%). Demographic data of the two groups are shown in Table 1. Both groups were comparable in terms of age at presentation, gestational age, and parity. The median age at presentation was 30 (interquartile range [IQR], 25-34) years. The median gestational age was 39 (IQR, 38-40) weeks.

**Table 1:** Demographic characteristics between study group and control group (n=934) Values are presented as mean ± standard deviation or number (%). ^a^Mann-Whitney U test was used to compare continuous variables,^ b^Pearson's Chi-Square was used to compare categorical variables. P-value < 0.05 is considered statistically significant.

Demographic characteristics	Sickle cell trait		P-value	Total n (%)
	Yes (n =460)	No (n = 474)		
Maternal age at delivery (years), mean ± SD	30± 6	30± 6	0.761^a^	934 (100)
Maternal age at delivery (years), n (%)			0.107^b^	934 (100)
<20	6 (1.3)	8 (1.7)		14 (1.5)
20-24	84 (18.3)	96 (20.3)		180 (19.3)
25-29	141 (30.7)	126 (26.6)		267 (28.6)
30-34	113 (24.6)	137 (28.9)		250 (26.8)
35-39	90 (19.6)	69 (14.6)		159 (17)
≥40	26 (5.7)	38 (8)		64 (6.9)
Gestational age at delivery, mean±SD	38±2	38±2	0.390^a^	934 (100)
Parity, mean±SD	3±1	3 ±2	0.163^a^	934 (100)

Overall, maternal complications were noted in 31.2% (n=291) which include PRHD, GDM, Operative vaginal delivery, and emergency cesarean sections, while newborn complications were found in 16% (n=149) which include SGA, PTD, and IUFD (Table 2). In univariate analysis, patients with SCT were significantly more likely to underwent cesarean sections compared to healthy women, 28.7% (n=132) vs. 21.7% (n=103), respectively (P=0.044) and to have more IUFD (3% (n=14) vs. 0.2% (n=1), respectively (P<0.0001). There were no statistical differences between types of cesarean sections, elective or emergency cesarean sections. Furthermore, the proportion with PRHD, GDM, SGA, and PTD were similar in both groups (Table 2).

**Table 2:** Comparison between adverse maternal and newborns outcomes in both groups (n=934) Values are presented as number (%) or mean ± standard deviation. ^a^Fisher’s exact test or ^b^Pearsons Chi-Square test were used to compare categorical variables, ^c^Mann-Whitney U test was used for continuous variables. P-value <0.05 is considered statistically significant.

Adverse pregnancy outcomes	Group A (n=460, 49.3%)	Group B (n =474, 50.7%)	P-value	Total n (%) (n=934, 18.4%)
Maternal outcome				
Pregnancy related hypertensive disorders	16 (3.5)	16 (3.4)	1.000^a^	32 (3.4)
Gestational diabetes	38 (8.3)	45 (9.5)	0.566^a^	83 (8.9)
Mode of delivery				
Vaginal delivery	321 (69.8)	361 (76.2)	0.044^b^	681 (73)
Operative vaginal delivery	7 (1.5)	10 (2.1)		17 (1.8)
Cesarean section	132 (28.7)	103 (21.7)		235 (25.2)
Elective cesarean section	43 (32.6)	33 (32)	1.000^a^	76 (8.1)
Emergency cesarean section	89 (67.4)	70 (68)		159 (17)
Newborns outcome				
Birth weight (grams), mean±SD	3089 ± 0.581	3095 ± 0.541	0.661^c^	
Preterm delivery	50 (10.9)	48 (10.1)	0.749^a^	98(10.5)
Small for gestational age	20 (4.3)	16 (3.4)	0.498^a^	36 (3.9)
Intrauterine fetal death	14 (3.0)	1 (0.2)	< 0.0001^a^	15 (1.6)

## Discussion

SCD is well known to be a high-risk condition as it demonstrates an increased risk of patient morbidity and mortality. It is also associated with an increase in pregnancy-related complications such as pre-eclampsia, stillbirth, PTD, and SGA infants [4]. On the other hand, while SCT is considered an asymptomatic carrier state, studies have shown that it is not a wholly harmless condition [17]. It has been associated with serious conditions, such as venous thromboembolism (VTE), chronic kidney disease (CKD) and exercise-related sudden death [7,12,17,18].

In this retrospective study, SCT occurred in approximately 16.4% of all patients delivered in SMC during the study period. The incidence is considered relatively high in comparison to other studies which reported a lower incidence of SCT patients [8,19]. Canelon et al. [19] and Larrabee et al. [8] reported that the incidence of SCT among parturition mothers is 3.9% in Pennsylvania and 10.2% among African American women, respectively. This can be explained by the higher incidence of SCT in Bahrain 14.7% as reported by the newborn screening service in 2010 [1].

The most noticeable finding in this study is the 15-fold increase in the risk of IUFD among women with SCT (3%) compared to women with normal hemoglobin (0.2%) (P<0.0001). Similar results were reported by Canelon et al.'s study [19]. This might be attributed to the sickling of maternal blood red cells at the placental blood vessels which may lead to decreased oxygenation, thrombosis, and placental infarctions causing intrauterine fetal hypoxia [20]. Tylor et al. [21] studied placental pathology and found sickling in intervillous space and decidual blood vessels in women with SCT. Also, they noticed more frequent ascending amniotic infection and meconium histiocytosis in the SCT group compared to the control group. The increased rate of ascending amniotic fluid infection in the SCT group compared to non-carriers by itself may adversely affect the fetus through microbial invasions and proinflammatory cytokines [22]. Subsequently, both mechanisms; sickling and infections, may contribute to our findings, and negatively impact the fetus causing severe neonatal morbidity and mortality [21,22]. Further well-designed prospective randomised studies need to confirm which of these hypotheses may abetted to our findings.

In the present study, the incidence of pregnancy-related hypertension did not defer between patients with SCT (3.5%) and those with normal hemoglobin (3.4%) (P=1.000). This finding coincides with a report by Tylor et al. [21]. However, Larabee and Monga's study [8] reported an increase in hypertensive disease among patients with SCT (P<0.0001).

Unlike Tan et al.'s study [23] which noted a difference in neonatal birth weight among offspring of pregnant mothers with SCT, our study did not find an increase in low-birth-weight offspring of this group of mothers. Regardless of their findings, they suggested that the higher SGA may be due to pregnancy complications or social deprivation in their study population. Tan et al. conducted another study [24] published in 2008 to determine the mean birth weight and mean customized birth weight centiles to analyze the risk of SGA among babies of SCT pregnancies in relation to maternal body mass index (BMI), ethnicity and parity, and the infant’s gender and gestational age. They found that the mean birthweight of offspring of SCT mothers is significantly lower by 57 g compared to healthy women, 3,223 ± 661 versus 3,280 ± 616 g, respectively (P=0.024). Nonetheless, they conclude that SCT is not a risk factor for SGA. This was in line with our findings where sickle cell carriers were not associated with increased risk of having SGA fetuses. the mean Yet, the birthweight difference between babies of mothers with SCT and those with normal hemoglobin was not statistically significant, 3,089 ± 581 versus 3,095± 541 g, respectively (P=0.661).

Limitations and strengths

This study has several limitations being a retrospective study which may lead to an ascertainment bias, particularly in the control group. Moreover, confounding variables that might affect maternal and newborn outcomes had not been accounted for such as maternal BMI and previous obstetrics history including past maternal and child outcomes as these data were not available from the existing registry book. Despite these limitations, this study has a relatively large number of patients and of the comparative groups (SCT and normal hemoglobin). Moreover, it has no missing data from the selected variables during extraction or analysis. Furthermore, the well-established universal screening for hemoglobinopathy program in Bahrain allowed us to find all patients with SCT delivered in the period of the study. This study is the first study conducted in Bahrain to analyze the adverse pregnancy outcome in this group of patients. Given the high prevalence of SCT carriers in Bahrain, our study can help in prenatal counseling and antenatal surveillance. This study is a significant contribution to the literature and its findings can be useful for any healthcare provider dealing with patients from high SCT prevalence countries.

## Conclusions

In conclusion, the data in this study have shown that other than the increase in IUFD, SCT is not associated with increased adverse pregnancy outcomes such as pregnancy-related hypertensive disorder, gestational diabetes, SGA, and PTD. However, we cannot ignore that the SCT is associated with a significant increase in IUFD. Therefore, it is reasonable to give attention to such findings which could be a trigger to initiate conducting well-designed studies to scrutinize these findings and to determine whether there may be a benefit of a unique antenatal surveillance guideline for such patients.
